# Vital Pulp Therapy of a Symptomatic Immature Permanent Molar with Long-Term Success

**DOI:** 10.22037/iej.2016.19

**Published:** 2016

**Authors:** Sedigheh Sabbagh, Alireza Sarraf Shirazi, Mohammad Jafar Eghbal

**Affiliations:** a *Student Research Committee, School of Dentistry, Mashhad University of Medical Sciences, Mashhad, Iran; *; b *Dental Material Research Center, School of Dentistry, Mashhad University ofMedical Sciences, Mashhad, Iran; *; c *Iranian Center for Endodontic Research, Research Institute of Dental Sciences, Dental School, Shahid Beheshti University of Medical Sciences, Tehran, Iran*

**Keywords:** Apexogenesis, Calcium-Enriched Mixture, Immature Tooth, Irreversible Pulpitis, Pulpotomy, Vital Pulp Therapy

## Abstract

Vital pulp therapy (VPT) is the preferred conservative treatment for preservation of symptomatic pulps in immature permanent teeth. The present case report summarizes VPT of an immature permanent molar with irreversible pulpitis associated with apical periodontitis in a 9-year-old boy. Cervical pulpotomy was performed and radicular pulp stumps were covered with calcium-enriched mixture (CEM) cement; the tooth was then restored with stainless steel crown. After a 50-month follow-up period, the pulpotomized molar was clinically functional and asymptomatic. Moreover, radiographic evaluation revealed evidence of complete root development as well as normal periodontal ligament around the roots. The successful outcome achieved through VPT using CEM biomaterial in the reported case suggests that this method may produce favorable outcome for vital immature permanent teeth with irreversible pulpitis and periapical disease.

## Introduction

Irreversible pulpitis is clinically characterized by persistent pain after removal of an external stimulus [1, 2]. If such an inflammatory reaction continues, periradicular tissues can consequently be affected. This can ultimately lead to development of apical periodontitis which, in the light of available evidence, can be accompanied by presence of a vital pulp [2]. It is generally accepted that root canal therapy (RCT) is the standard treatment for irreversible pulpitis when economic issues are not a matter of importance. However, the survival rate of endodontically treated teeth compared with vital teeth is not promising, particularly in molars [3].

In modern dentistry, vital pulp therapy (VPT), whenever feasible, should be considered as an effective reasonable alternative to RCT [1]. In clinical practice, VPT includes stepwise excavation, pulp capping (direct and indirect) and pulpotomy (miniature, partial and complete), based on the amount of preserved coronal pulp [4]. The main goal of all these conservative methods is to maintain the normal pulp or remove contaminated pulpal tissue in order to promote the soft tissue repair process. [5]. Therefore, immature teeth eventually gain additional benefit from VPT, considering their weak structural nature [5].

Calcium-enriched mixture (CEM) cement is used in different VPT techniques [6]. This tooth-colored water-based endodontic biomaterial is a biocompatible mixture mainly consisting of a variety of calcium compounds. CEM cement is potentially capable of forming hydroxyapatite (HA) by using indigenous sources from which calcium and phosphate ions are released. The produced HA then induces dentinal barrier formation [6, 7]. Moreover, CEM biocement has the ability to induce differentiation of human dental pulp stem cells [8].

This report presents the successful management of irreversible pulpitis and concurrent apical periodontitis in an immature permanent molar through cervical pulpotomy using CEM cement.

**Figure 1:**
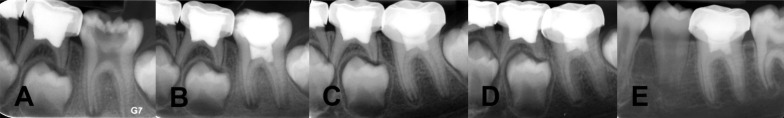
A) Pre-operative intraoral radiograph. Note the extensive caries in lower left permanent molar, open apices and an apical lesion in mesial root*; B)* Immediate postoperative radiography; *C)* Seven-month follow-up; *D*) Recall after 15 months: note the normal periodontal ligament (PDL) space and root development *E*) Fifty-month recall radiography showing complete root formation with normal PDL space

## Case Report

A 9-year-old boy with a symptomatic first mandibular left permanent molar was referred to a private dental clinic. The patient complained about spontaneous and lingering pain provoked by chewing or cold drink. His medical history revealed no serious systemic conditions. Intraoral examination was performed: there was an extensive occlusal carious lesion on the lower left first permanent molar. Nonetheless, the surrounding periodontal tissues were intact except for mild marginal gingivitis and there were no visible signs indicating pulp necrosis, namely swelling, draining fistula or excessive mobility. The tooth responded to pulp vitality test with sever lingering pain and was sensitive to percussion. Additionally, preoperative periapical radiographies showed immature roots with apical radiolucency (Figure 1A). Based on the clinical and radiographic evaluations, the final diagnosis was symptomatic irreversible pulpitis associated with apical periodontitis.

VPT for the involved molar was instructed. After achieving profound anesthesia using 2% lidocaine with 1:80000 epinephrine (Darupakhsh, Tehran, Iran) for inferior alveolar nerve block the tooth was isolated. After caries excavation, pulp chamber was unroofed and coronal pulp was completely removed using a sterile high-speed diamond bur (Diatech, Heerbrugg, Switzerland) under water coolant. Post-amputation hemorrhage was controlled with a sterile cotton pellet soaked with 5.25% NaOCl and placed on the pulp stump with gentle pressure for 5 min. Next, following the manufacturer’s instructions, CEM cement (BioniqueDent, Tehran, Iran) powder and liquid were mixed together and introduced into the pulp chamber using a sterile plastic instrument. A sterile dry cotton pellet was then used to gently adapt the biomaterial to the clot free pulpal wounds and cavity walls, and simultaneously to remove excess moisture from the cement. Finally, the tooth was restored with composite resin and a standard post-operative radiography was taken (Figure 1B). After one week, the pulpotomized tooth was clinically re-examined. The tooth was symptom-free and, therefore, coronal restoration was completed with stainless steel crown.

At the 7-, 15- and 50-month follow-up visits, the treated molar clinically remained asymptomatic and periapical radiographies showed complete healing of the apical lesion and complete root development (Figure 1C, 1D and 1E). In addition, calcified bridges were clearly observed on 7-month post-treatment radiography in both mesial and distal roots (Figure 1C).

## Discussion

Pulp involvement is the inevitable consequence of leaving progressing dental caries untreated [9]. Irreversible pulpitis, as the advanced phase of vital pulp disease, is regarded as an indication for RCT [9, 10]. Concerning immature permanent teeth, such treatment can be challenging. This is justified as a result of their immature apices which compromise obtaining apical seal, and maintaining the obturating material within the canal space. Furthermore, thin root dentin makes these young teeth more prone to fracture [11]. It is widely acknowledged that efforts should focus on preserving pulp vitality when dealing with immature permanent teeth, thereby VPT is the current treatment of choice. As a result, root development will further continue and thickening of dentinal root walls will consequently strengthen the root(s) against fracture [12]. In addition, the major advantages of VPT over RCT, when considering pediatric patients, may include reduced time, lower cost and relative simplicity of the procedure, less post-operative pain as well as reduced number of radiographies taken throughout the treatment [1, 3, 10].

In the present case report, a first permanent immature molar diagnosed with irreversible pulpitis and apical periodontitis was treated with cervical pulpotomy using CEM bioceramic. Long-term evaluation clearly indicated pulp vitality, full root development as well as healed apical lesion. Besides, root development in addition to apical closure has been stated to be the most reliable prognostic criteria for assessing success of VPT in an immature permanent tooth [13]. In the reported case, both of the aforesaid indicators were present after the long term follow-up period. Therefore, the treatment was judged a success.

Among published literature, there are several human studies on successful VPT of mature permanent teeth diagnosed with irreversible pulpitis, using CEM cement [2-4, 14]. Otherwise, there is little high-level clinical evidence regarding conservative pulp treatment using CEM biocement in immature permanent teeth with the same pulp condition; Nosrat *et al*. [15] conducted a randomized clinical trial in which clinical and radiographic outcomes of apexogenesis with MTA and CEM cement in carious-exposed vital immature molars were blindly evaluated. Over 12-month follow-up, symptomatic cases comprising the majority (67%) of selected teeth showed a success rate of 100% following pulpotomy treatment. Recently, a clinical study by Peng *et al.* [16] investigated the short-term outcomes of partial/complete pulpotomy using MTA in immature permanent teeth with irreversible pulpitis. One-year success rate was calculated as 91%. Moreover, dentine bridge formation was radiographically seen in 65% of the cases. Harandi *et al*. [13] compared the different pulpotomy materials, *i.e.* ZOE, MTA and CEM cement, on three immature teeth of a person diagnosed with established irreversible pulpitis and normal periapical conditions. After 18 months revealed clinical success as well as apical closure of all treated teeth. However, in the case of ZOE, a slight PDL widening was observed. Another case report described CEM apexogenesis treatment of an immature symptomatic molar. The pulpotomized tooth was clinically symptom-free at all follow-up assessments and 12-month radiographic examination showed full root development as well as calcified bridge formation [17].

Nowadays, bioceramics are widely considered to be suitable alternatives for traditionally-used calcium hydroxide in VPT [5]. Mineral trioxide aggregate (MTA) and CEM cement are obvious examples of such biocements. Concerning complete pulpotomy, it has been shown that both MTA and CEM cement are similar in terms of underlying pulp status and calcified bridge formation, and both significantly better than calcium hydroxide in this regard [18]. Both cements display similar and comparable sealing ability, setting expansion and cytocompatibility [6]. However, CEM is generally superior in setting time, flow, film thickness, handling and antibacterial characteristics to MTA [19, 20]. Finally, pulp healing promoted by CEM cement may be attributed to sealing capacity, antimicrobial activity, insignificant cytotoxicity, similarity to dentin and induction of hard tissue barrier [2].

## Conclusion

In the present case report, successful preservation of pulpal vitality and function was achieved using CEM cement for VPT of an immature permanent molar with concurrent irreversible pulpitis and apical periodontitis. 
